# Super-Adsorptive Biodegradable Hydrogel from Simply Treated Sugarcane Bagasse

**DOI:** 10.3390/gels8030177

**Published:** 2022-03-14

**Authors:** Md. Ibrahim H. Mondal, Md. Obaidul Haque, Firoz Ahmed, Md. Nahid Pervez, Vincenzo Naddeo, Mohammad Boshir Ahmed

**Affiliations:** 1 Polymer and Textile Research Lab, Department of Applied Chemistry and Chemical Engineering, Rajshahi University, Rajshahi 6205, Bangladesh; obd_haq@yahoo.com (M.O.H.); fahmed0902@bcsir.gov.bd (F.A.); 2 BCSIR Laboratories Rajshahi, Bangladesh Council of Scientific and Industrial Research, Rajshahi 6206, Bangladesh; 3 Sanitary Environmental Engineering Division (SEED), Department of Civil Engineering, University of Salerno, Via Giovanni Paolo II 132, 84084 Fisciano, Italy; mpervez@unisa.it; 4 Bio-Electronics Materials Laboratory, School of Materials Science and Engineering, Gwangju Institute of Science and Technology (GIST), Gwangju 61005, Korea; mohammad.ahmed@gist.ac.kr

**Keywords:** sugarcane bagasse, hydrogels, water absorption, biodegradable, hydrophilic

## Abstract

There is a great demand for biodegradable hydrogel, and cellulose enriched wastes materials are widely used to serve this purpose for various advance applications (e.g., biomedical and environmental). Sugarcane bagasse is cellulose-enriched agro-waste, abundantly grown in Bangladesh. This study aimed to treat sugarcane bagasse-based agro-waste using a sustainable and ecofriendly approach to produce hydrogel with super-swelling capacity for adsorption of copper, chromium, iron ions, methylene blue and drimaren red dyes. To increase the swelling property of hydrogels, copolymerization of hydrophilic monomers is an effective technique. Therefore, this study aimed to prepare hydrogel via free radical graft-copolymerization reaction among acrylamide, methyl methacrylate and treated bagasse in the presence of N,N-methylene-bis-acrylamide as a crosslinker and potassium persulphate as an initiator. To obtain maximum yield, reaction conditions were optimized. It was found that hydrogel obtained from chemically treated sugarcane bagasse showed maximum water absorption capacity of 228.0 g/g, whereas untreated bagassebased hydrogel could absorb ~50 g/g of water. Maximum adsorption capacity of 247.0 mg/g was found for copper ion. In addition, organic pollutant removal from industrial effluent also showed good performance, removing >90% of methylene blue and 62% of drimaren red dye, with shorter kinetics. The biodegradability study showed that after 90 days of exposure, the hydrogels degraded to about 43% of their own mass. Therefore, the produced hydrogel could be an alternative adsorbent to remove pollutants and also for other potential applications.

## 1. Introduction

Hydrogels are hydrophilic polymeric materials that swell in the presence of water without dissolving [1,2]. They are three-dimensional cross-linked polymeric macromolecules and are also termed as superabsorbent polymers. Hydrogel materials are synthesized via two main approaches: chemical or physical crosslinking [3]. Hydrogels are more susceptible to polar solvents like water due to the presence of inherent polar functional groups (e.g., –OH, –COOH, –CONH_2_, –CONH, –SO_3_H, etc.) in their backbone and remain insoluble, as they are mainly prepared in the form of cross-linked networks [4]. However, their swelling ratios depend on the structure of the polymeric network itself and also on the environmental factors such as temperature, pH and ionic strength of the media [5]. Hydrogels are widely used in various advanced applications, such as personal hygiene products, underwater devices, bioelectronics device development, water reservoirs for dry soils and environmental applications [6,7,8,9,10]. In addition, hydrogel has many advanced applications, such as biomedical device development, including soft contact lenses, lubricating surface coatings, phantoms for ultrasound-based imaging, controlled drug release devices, wound dressings for healing, cell immobilization islets, three-dimensional cell culture substrates, and bioactive scaffolds for regenerative medicine [11,12,13,14,15,16,17,18,19].

There is a great demand for the eco-friendly preparation of biodegradable hydrogels from natural materials. For this purpose, incorporation of natural polymer such as cellulose, starch, chitosan, proteins, carrageenan, etc., in the polymeric chain to produce hydrogels for various purposes have been practiced [20]. Among them, cellulose is the most abundant and most used ubiquitous and biodegradable natural polymer; it is used in preparing such kinds of hydrogels [21,22,23,24]. To extract cellulose, different lignocellulosic substances, such as wood sawdust, crop milling waste, sugarcane bagasse, bamboo plant waste and other ligno-cellulosic sources, are being used for hydrogel preparation. Among them, sugarcane bagasse is an agro-waste that contains cellulosic material ~43%, and it has great potential to produce hydrogel [22,23,24,25,26,27,28,29]. To prepare hydrogel from sugarcane-based cellulosic materials, different approaches have been used [26,28]. In general, two common routes were followed for the synthesis of cellulose-based hydrogels: (i) graft copolymerization of hydrophilic vinyl monomers onto cellulose and its derivatives and (ii) crosslinking by functional monomers onto cellulose and its derivatives [22,24,30,31,32,33,34,35,36,37,38]. For example, recently, Lu et al. [25] produced nanocellulose hydrogel based on sugarcane bagasse cellulose, and they followed a reported method using a complex and TEMPO-mediated oxidation process. On the other hand, Maity et al. [26] used acrylamide, acrylic acid, N, N′-methylene bis acrylamide and sugarcane bagasse-based extracted cellulose to produce hydrogel by a free radical solution polymerization technique for copper ion removal; however, their produced hydrogel did not have enough swelling capacity (i.e., maximum 600%). Another study by Nakasone et al. [27] used pre-purification of cellulose followed by a phase inversion complex process to produce cellulose-based hydrogel film. Similarly, Chanklinhorm et al. [28] produced sugarcane bagasse and polyacrylamide-based hydrogel composite using a gamma irradiation technique for control release of urea fertilizer. Therefore, most of these and other studies for cellulose-based hydrogel preparation from sugarcane bagasse were concerned about the extraction and purification of cellulose and also followed complex steps of synthesis. However, there is not a simple method available for treatment of sugarcane bagasse biomass or other biomass to produce a super-hydrogel with higher swelling ratio and higher adsorptive performance.

Therefore, in this study, we aimed to produce sugarcane bagasse-based hydrogel using a simple pretreatment of the bagasse in a sustainable and economical way. For this reason, we treated sugarcane bagasse biomass and used it directly to produce hydrogel by a simultaneous graft copolymerization technique. The hydrogel preparation conditions were optimized through various different parameters, such as temperature, monomer concentration, and initiator and crosslinker concentration to produce higher water absorption–capable super adsorbent. Finally, we applied the prepared hydrogel for different inorganic ions and organic dye removal from water and industrial effluents.

## 2. Results and Discussion

### 2.1. Preparation of Bagasse-Based Cellulose-g-poly (AM-co-MMA) Hydrogels

**Figure 1:**
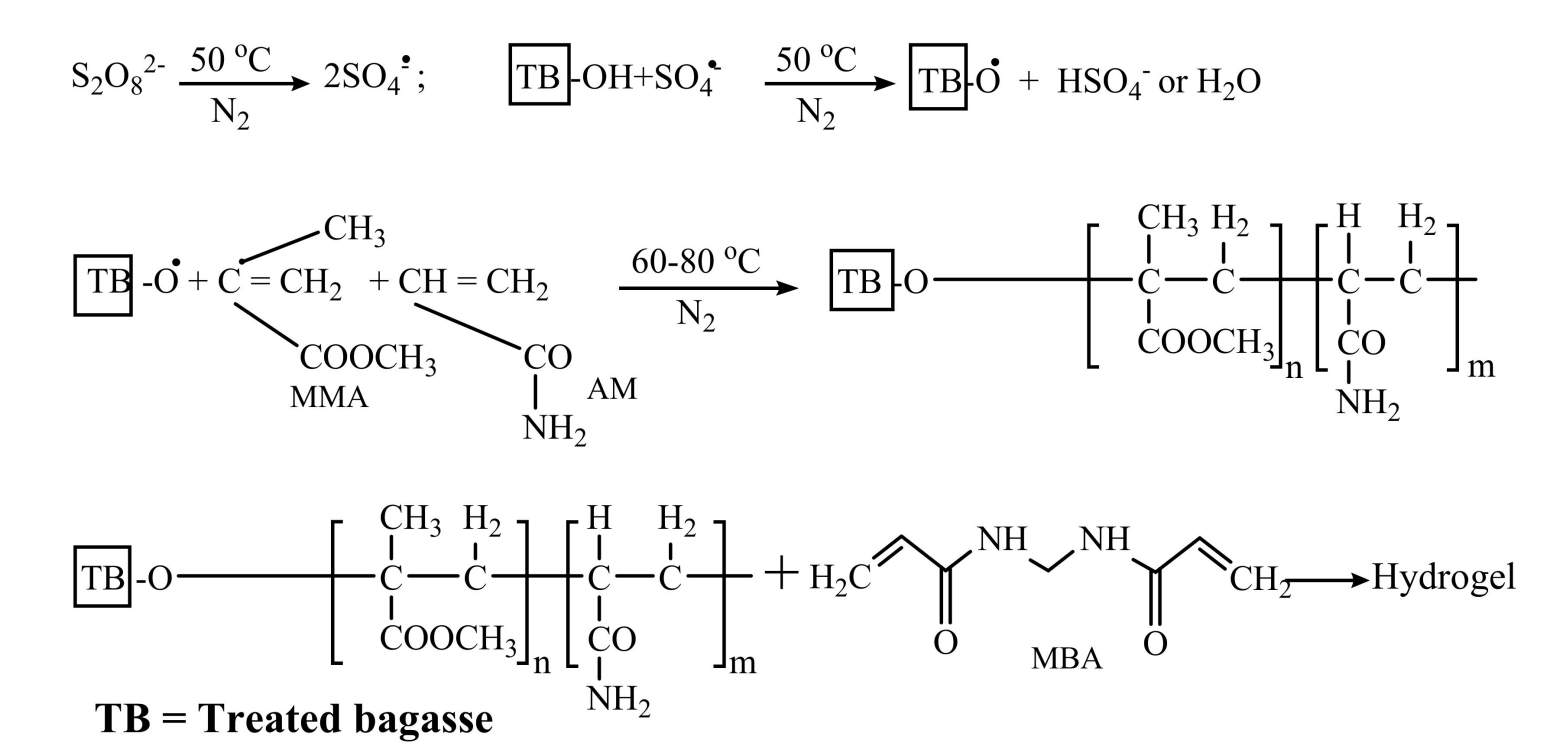
Reaction mechanism for the preparation of treated bagasse-based g-poly (AM-co-MMA) hydrogel [39,40].

Hydrogel was synthesized by graft copolymerization of acrylamide and methylmethacrylate onto bagasse, where KPS acted as a free radical initiator and MBA was used as a crosslinking agent. The following reaction might have happened during hydrogel preparation:

From Figure 1, it can be seen that KPS produced sulfate anion radicals during the heating and agitation stage. Once the sulfate anion radicals interact with the hydroxyl groups of cellulose fractions of treated bagasse, they become more active macroradicals (TB-O free radicals). The macroradicals (TB-O free radicals) then attack the monomers (i.e., AM and MMA) to form copolymer as well as propagate the polymeric chain reaction. Thereafter, they donate free radicals to neighboring molecules, which further enlarges the grafted chain. At the same time, the enlarged polymeric chain reacts with MBA to form a crosslinked structure (hydrogel) [39,40,41,42].

### 2.2. Influence of Different Parameters on Reaction Conditions of Synthesis Hydrogel

**Figure 2:**
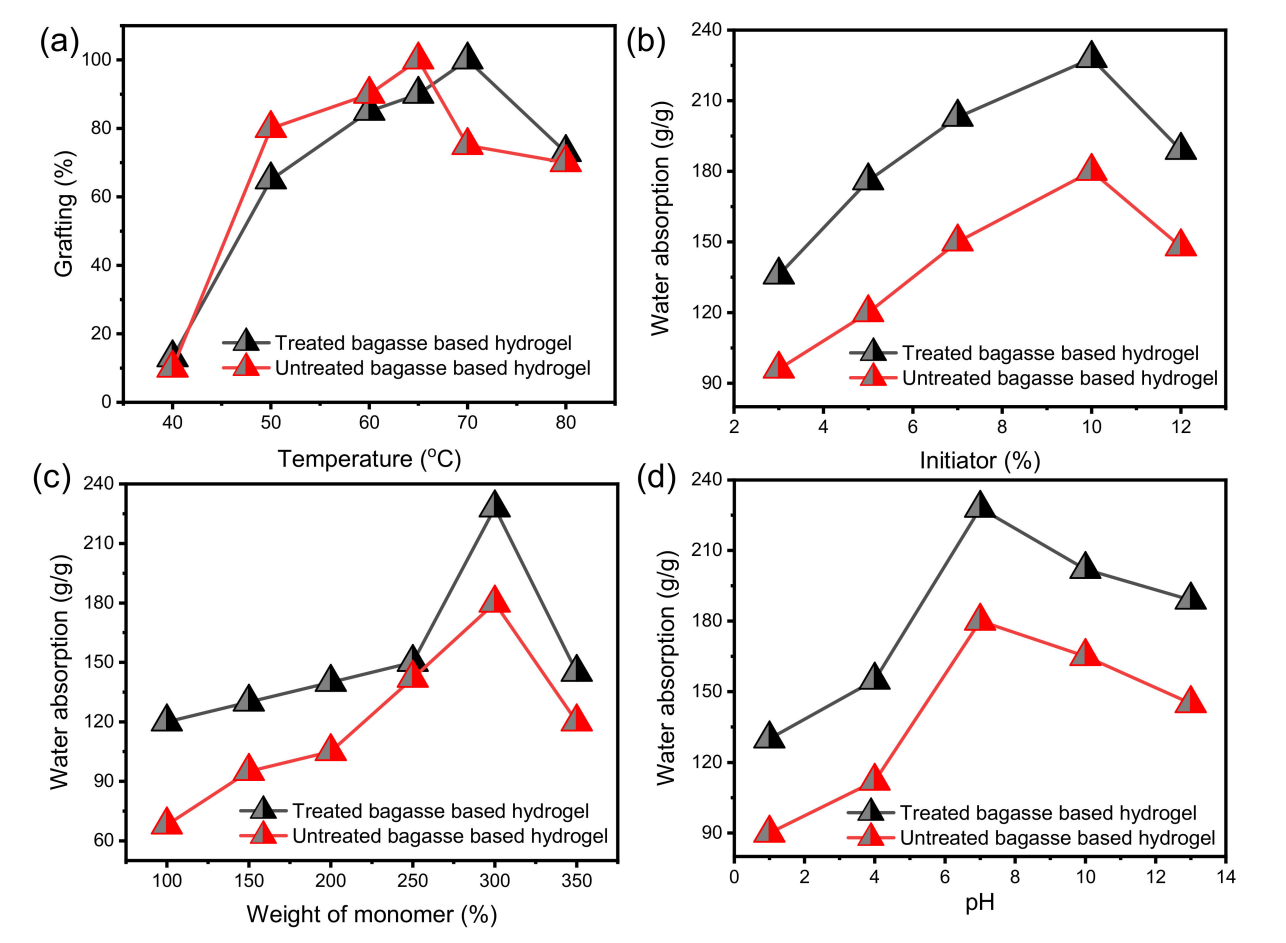
The influence of different reaction conditions on synthesis of hydrogel: (**a**) temperature, (**b**) concentration of initiator, (**c**) concentration of monomers, and (**d**) pH.

To achieve the desired properties in the hydrogel, we carried out optimization by controlling different parameters, such as variation of reaction temperature, initiator concentrations, monomer concentration and pH of the solutions. Figure 2a shows that with the increase of temperature to 65 °C, the grafting percentages were increased to ~100% for both treated and untreated bagasse-based hydrogel.

Figure 2b shows the relation between the water absorbency of a hydrogel and initiator concentration. It is clear that with the increase of initiator concentration up to 10%, the water absorption capacity increased linearly and then decreased, which indicated the optimum initiator concentration both for treated and untreated bagasse-based hydrogels. It is well-known fact that a free-radical polymerization reaction is strongly regulated by initiator concentration, which also plays a vital role in proper crosslinking [43]. However, after desired concentration of the initiator, it has negligible effects.

Figure 2c correlates the effect of monomer connection on the water absorbency of hydrogels. The results indicate that with the increasing of monomer concentration up to 300% (dry mass), water adsorption capacity increased significantly, which indicates the presence of more hydrophilic functional groups in hydrogels. Further increasing the monomer concentration leads to a decrease in water adsorption capacity, which indicates the unavailability of the monomer to hold more MMA and AM on the backbone of treated and untreated bagasse, leading to lower water absorbency [44]. The highest water absorbency was found to be 228 g/g for treated bagasse-based hydrogel, while a water absorbance capacity value of 180 g/g was found for untreated bagasse-based hydrogel.

Figure 2d shows the effect of pH on the swelling of hydrogels. Water absorption capacity was highest at pH 7 (a neutral medium). In neutral medium, the presence of hydrophilic ions (−COO^−^, −CONH_2_) was responsible for maximum swelling of hydrogels. Water absorption capacity was less in both the strong alkaline and acidic regions. The main reason behind this is that the swelling of hydrogel is mainly governed by the electrostatic repulsions between available ions present in the solution and the presence of functional groups on hydrogels. More clearly, at lower pH, i.e., in an acidic medium, the ionization of ester groups remains minimal in the presence of anions, leading to electrostatic repulsive force development, and therefore lower swelling ration was obtained. However, the highest water absorption capacity of the hydrogel was observed at pH ~7, where repulsion between anion and cations was minimal [45]. In all cases, treated bagasse-based hydrogel had more absorptive properties compared to untreated bagasse-based hydrogel. The main reason for this might be due to the removal of unwanted materials and the exposure of more surface-active functional groups in treated bagasse to facilitate co-polymerization for hydrogel preparation. More clearly, hemicelluloses are found on the cellulose fibril surface and bind the cellulose together with lignin. That is why the cellulose molecules are assembled into layers with systematic order and packed together into larger fibers. When sugarcane bagasse is subjected to acid hydrolysis, the systemic order and compactness of cellulose are destroyed due to the removal of hemicellulose and lignin, which ultimately expose more and more reaction sites on the cellulose backbone. Though hemicelluloses are the most hydrophilic, the acid treatment of bagasse is required for better crosslinking as well as swelling performance. These characteristics eventually helped to create super-performance in terms of water absorption for the treated bagasse-based hydrogel. Moreover, as the hydrogels respond differently in different pH, these hydrogels can also be referred as pH-sensitive hydrogels.

### 2.3. Characterizations and Morphology of Synthesized Hydrogel

**Figure 3:**
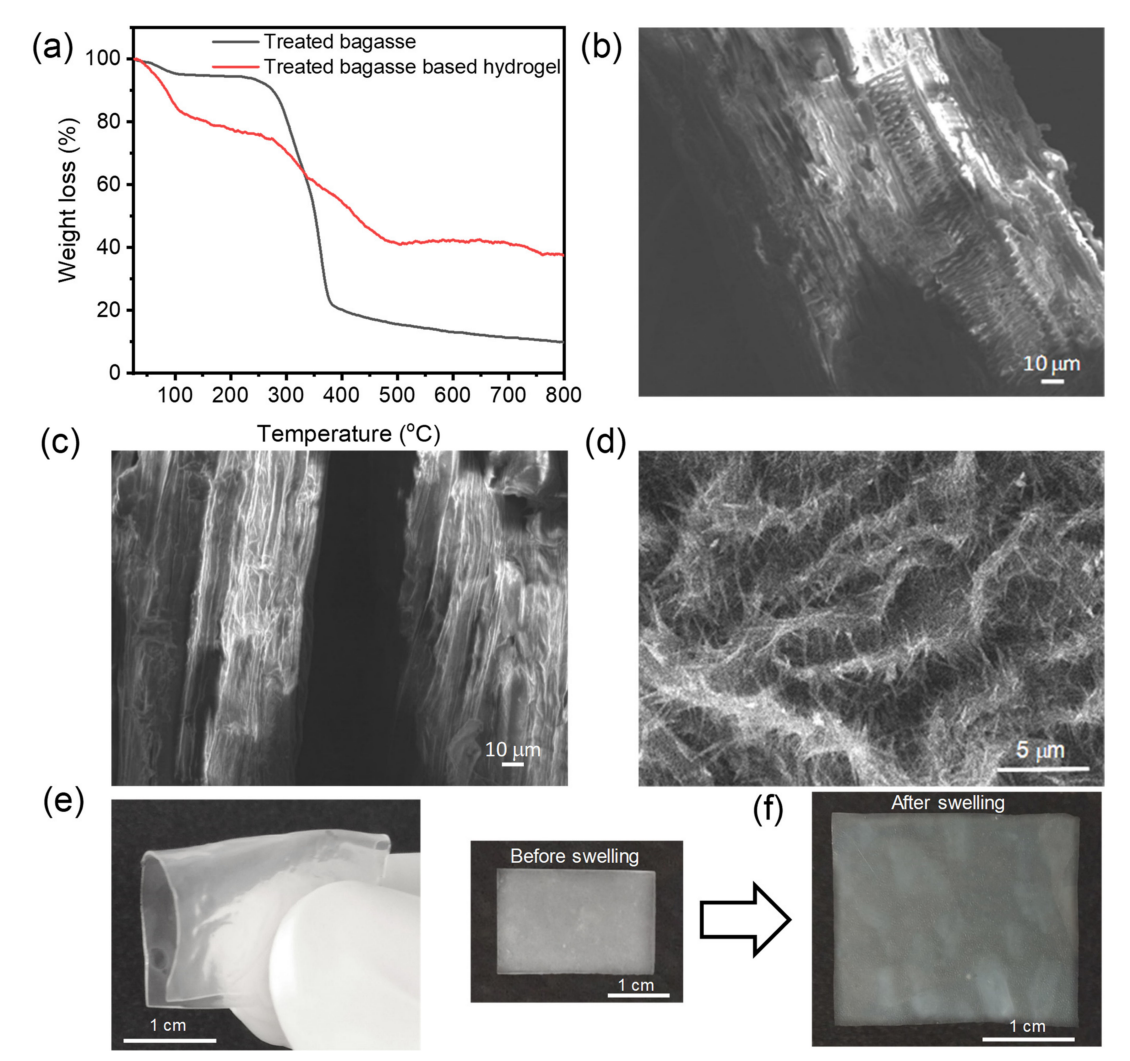
TGA curve of treated bagasse and its hydrogel (**a**). SEM images of sugarcane bagasse (**b**); treated bagasse (**c**); hydrogel (**d**); hydrogel film for flexibility test (**e**); and swelling capacity of the hydrogel (**f**).

Thermogravimetric analysis (TGA) provides in-depth information on the moisture content, release of different fractions, i.e., degradation fractions at different temperatures, and some structural information. Therefore, we carried out a TGA analysis of treated bagasse and hydrogel to determine the degradation pattern as well as the structural stability of the materials (Figure 3a). Treated sugarcane bagasse showed three stages of well-defined degradation areas, while the same degradation pattern was not observed for hydrogel prepared from treated bagasse. From thermal studies of cellulosic materials, in the first stage of degradation, bounded water and moisture are removed, and in the second stage, decomposition of cellulose takes place with the release of gaseous products; finally, the residue is found [39,41]. For treated bagasse, degradation continued up to ~375 °C, although a pronounced weight loss was observed at ~260 °C; at the end, ~12% weight was found as a residue when temperature rose to 725 °C. This is a similar pattern to that found in cellulose degradation by TGA. Hence, treated bagasse contained a greater fraction of cellulose. In parallel, weight loss in hydrogel continued up to ~ 475 °C, and ~58% of the substance was degraded. From the two curves, it can also be observed that the hydrogel had more thermal stability than treated bagasse. In addition, the co-polymerization technique provided a strong network structure, which led to more stability in thermal treatment. These eventually led the presence of a higher amount of ash/charcoal in the final mass. However, the presence of unwanted inorganics might also contribute to overall residual mass content.

To determine the morphology of the treated bagasse, untreated sugarcane bagasse, and produced hydrogel, we carried out SEM analysis (Figure 3b–d). A rough and non-porous rod-like surface of sugarcane bagasse appeared (Figure 3b), while treated bagasse appeared as a more porous structure, indicating many other fractions (i.e., impurities) were removed from the original bagasse mass (Figure 3c). On the other hand, for hydrogel based on treated bagasse, a continuous polymeric surface with small needle-like whiskers appeared, indicating the presence of many porous structures (Figure 3d). The tiny uneven surfaces of hydrogel contain intermolecular gaps that may help to uncoil polymeric networks and water diffusion during swelling of the hydrogel; therefore, hydrogel should have a high-swelling capacity. Therefore, our co-polymerization technique was very effective in producing a stable and high-swelling capacity hydrogel by a simple pre-treatment of biomass. This process does not require total purification of cellulose fractions from bagasse, therefore significantly reducing the chemical consumption. This should have added advantages as a greater amount of treated bagasse, i.e., higher yield, can be obtained, leading to a higher yield of hydrogel. Moreover, we took some physical photographs of the hydrogel samples (Figure 3e,f). From Figure 3e, it can be seen that the produced hydrogel film has excellent mechanical stability and softness capability. This can be confirmed due to the excellent bendability of the film (90°). Finally, the initial and swollen film also shows the stability of the hydrogel film (Figure 3f).

**Figure 4:**
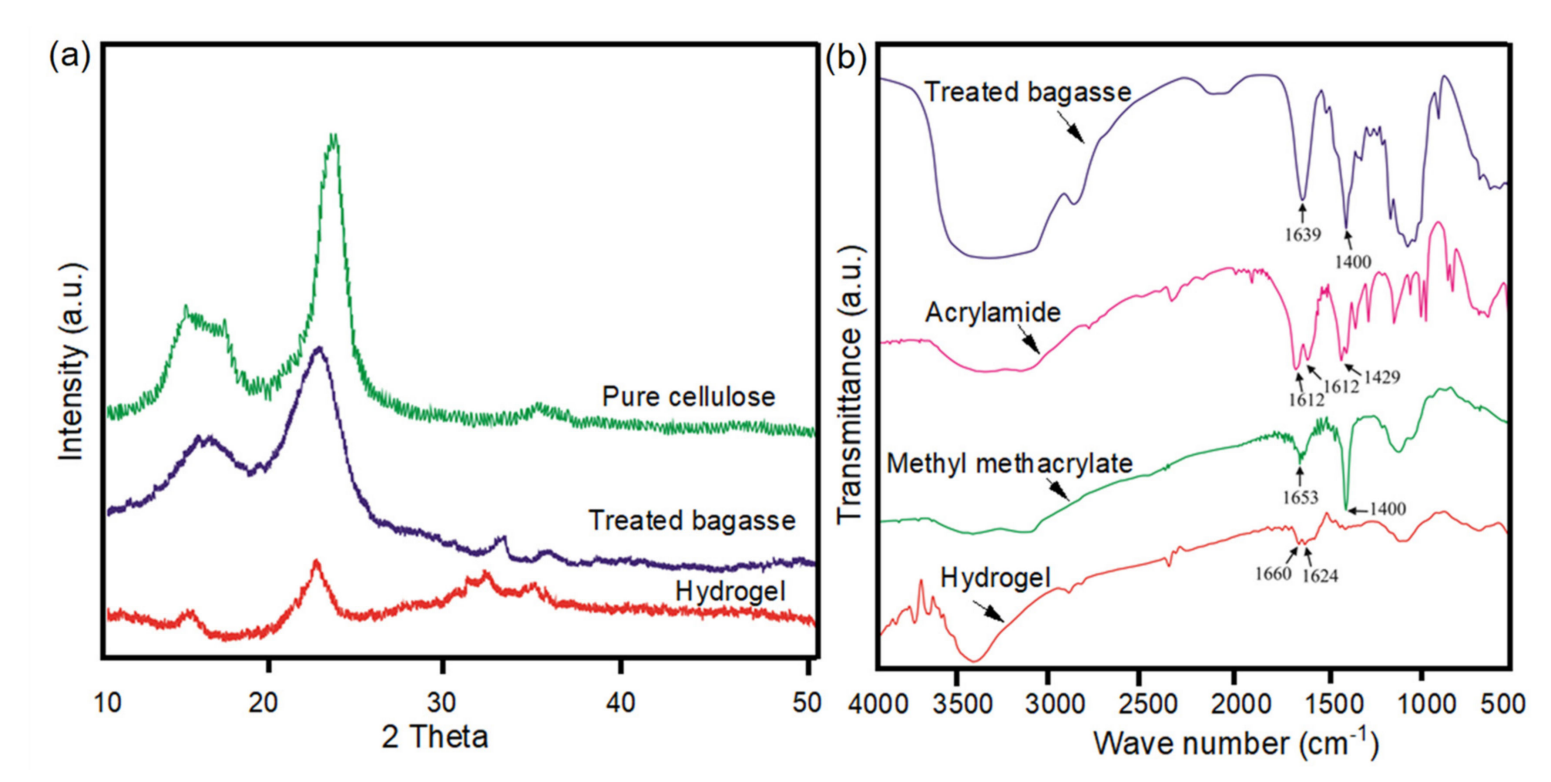
X-ray diffraction pattern of pure cellulose, treated bagasse and its hydrogel (**a**). FTIR spectra of treated bagasse, acrylamide, methyl methacrylate and hydrogel (**b**).

To check the orientation of the materials, we further carried out XRD analysis (Figure 4a). Three main characteristic broad peaks, at ~15°, 22–23° and 32–34.5° diffraction angles, can be observed for all materials. It is well known that sharp peaks indicate higher crystallinity, whereas broad peaks indicate amorphous regions. From the figure, it can be seen that higher crystalline main peak at 23° was obtained for pure cellulose, whereas this peak intensity was slightly broadened for treated bagasse. This study also indicates that our treated bagasse fraction was not in the full form of cellulose. Hence, due to the presence of other fractions, a slightly broader peak appeared. An even more pronounced difference was obtained for treated bagasse-based hydrogel material. This is reasonable, as the crystallinity of cellulose or treated bagasse was hampered due to the reaction of hydrophilic parts (i.e., –OH groups) with the monomer during hydrogel formation through the co-polymerization technique. Therefore, hydrogel formation from treated bagasse by polymerization reaction significantly reduced the crystallinity. In addition, we observed a similar trend in SEM images, i.e., the increase of porosity in treated bagasse and treated bagasse-based hydrogel.

We then carried out FTIR analysis to check the presence of different functional groups in the samples. The FTIR spectra of monomers, treated bagasse and synthesized hydrogel are shown in Figure 4b. A comparison between the treated bagasse and treated bagasse-based hydrogel provides insight into the reaction that took place between them. It is clear from Figure 4b that a peak at around 3500 cm^−1^ refers to the presence of –OH groups. This became boarder in the case of hydrogel. This indicated that the cellulose-based –OH groups took part in the reaction and produced a stable hydrogel structure. Two adjacent FTIR spectra (for Figure 4b), at 1660 and 1624 cm^−1^, can be assigned to the characteristic absorption peaks of the carboxamide-functional groups of amide moiety of the AM unit, and C=O asymmetric stretching in the carboxylate anion, respectively. On the other hand, a sharp peak at 1639 cm^−1^ representing C=O stretching appeared for treated bagasse. However, in the hydrogel, some of the characteristic peaks of individual molecules, such as MMA, AM and treated bagasse, did not appear; however, a small change in the shape and position of peaks was observed. These results indicate that treated bagasse-based –OH functional groups took part in the reaction to produce hydrogel via grafting reaction among AM, treated bagasse and MMA monomers.

### 2.4. Water Absorption Capacity, Biodegradability of Hydrogel and Gel Content in Hydrogel

**Figure 5:**
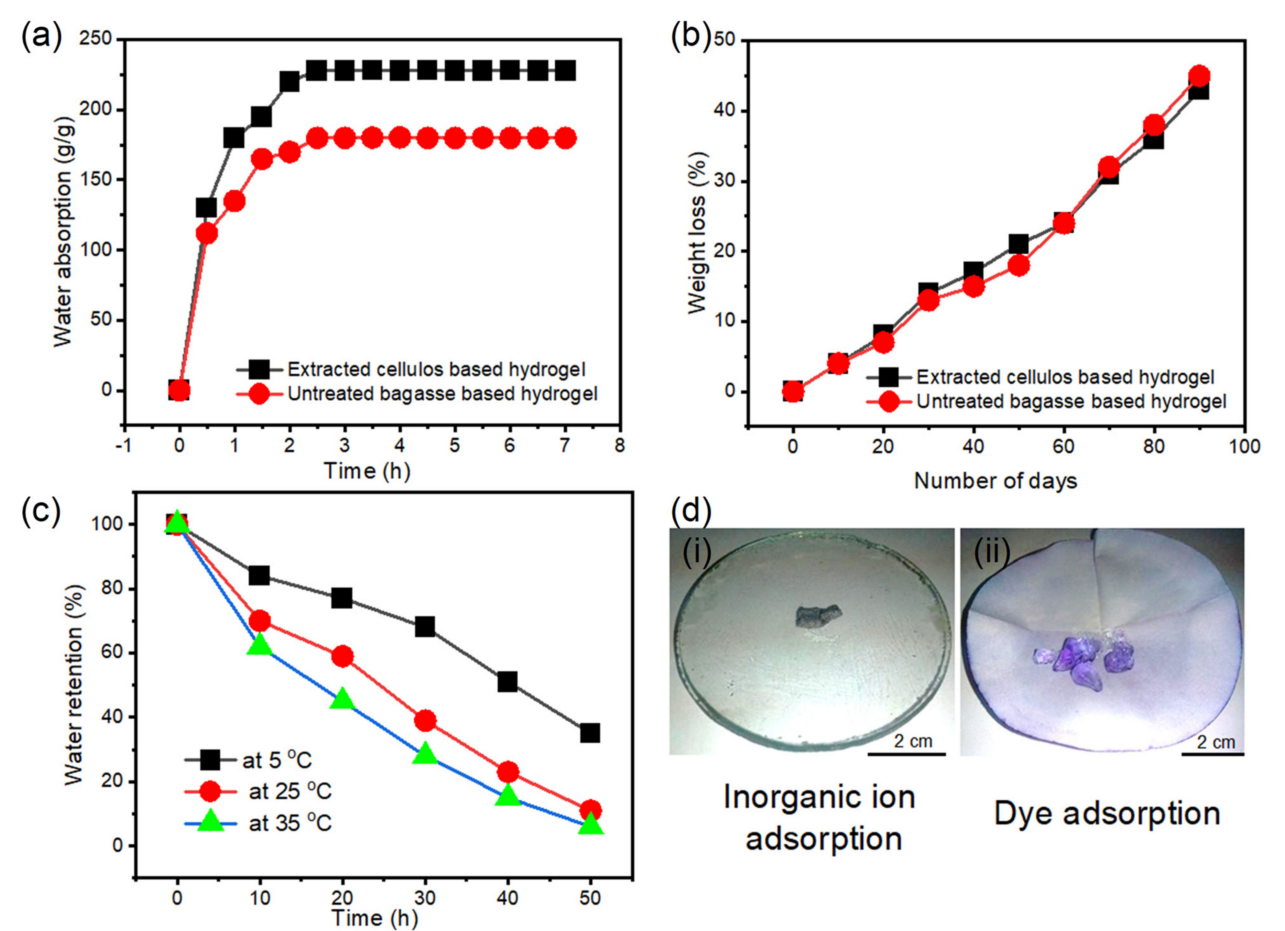
Time required for equilibrium water absorption (**a**); biodegradation scenario of hydrogel (**b**); water retention capacity of hydrogels at different temperatures, such as 5.0 °C, 25.0 °C, and 35.0 °C (**c**); adsorption performance of inorganic (**d**(**i**)) Cr ion and (**d**(**ii**)) dye from industrial effluent by hydrogel.

Figure 5a shows the equilibrium water absorbance capacity of the hydrogels. From the graph, it is clear that treated bagasse-based hydrogel had higher water absorbance capacity (230.0 g/g) compared with untreated bagasse-based hydrogel (172.5 g/g). It is noticeable that after 2 h of exposure, the absorbance values increased to the pinnacle for both hydrogels. Hence, only 2 h was required to achieve the highest swelling ratio for our hydrogels. Figure 5b shows the biodegradable nature of the hydrogels. It can be seen that with the increase of biodegradation time, the degradation of hydrogel increased almost linearly. About 43% of the weight of the hydrogel could degrade after burial in soil for 90 days, while untreated bagasse-based hydrogel degraded at a slightly faster rate (~45%). This might be lack of bonding of monomers with bagasse during hydrogel formation. Therefore, produced hydrogels had higher degradation in soil.

Gel fraction is the indication of the degree of crosslinking in the hydrogel. If the degree of crosslinking is high, a strong gel results, makes the structure rigid and reduces the ability of hydrogel to absorb water. The transition from a system with finite branched polymer to infinite molecules is known as sol-gel transition. Hydrogel is a sol-gel transition. When sol is removed from hydrogel, the remaining portion of the hydrogel is called the gel fraction of hydrogel. The higher the gel fraction, the better the quality of the hydrogel [46]. The gel content of the synthesized hydrogel was 90.1%, indicating the stronger hydrogel formation (Table 1).

### 2.5. Water Retention Capacity of Hydrogels

Recent application of hydrogel includes gradual release of water as well as nutrients like fertilizer and pesticides in agriculture. In that sense, the water retention capacity of hydrogel is an important property when the hydrogel will be used as an absorbent. Figure 5c shows that synthesized hydrogel can hold water for about two days at low temperature. Water release data were calculated at three different temperatures, i.e., at 5, 25 and 35 °C. It was found that an increase of temperature for the test resulted in decreases of water holding capacity due to rapid evaporation and breakdown of hydrogen bonds of water molecules [47,48].

### 2.6. Adsorption of Metal Ions and Dyes

The hydrogels were applied to adsorb metal ions and dyes from aqueous solutions. For this, aqueous solutions of three ions, i.e., Cu^2+^, Cr^3+^ and Fe^2+^, with a concentration of 50 ppm, were prepared. A small amount (about 0.10 g) of dry hydrogel sample was immersed into the beaker containing (100 mL) of each individual ionic solution. After stirring for 3 h, the sample became colored, which indicates that the hydrogels had adsorbed colored ions from the solution. The adsorption capacities of ions by the hydrogel were 247 mg/g, 240 mg/g and 57 mg/g, respectively, for Cu^2+^, Cr^3+^ and Fe^2+^.

A similar procedure was followed for the adsorption of two dyes. For this purpose, we collected the industrial effluent containing drimaren red (DR) and methylene blue (MB) from Sopura silk industry, a local industry. As the raw effluent is highly concentrated, it was combined with a mixture of various compounds. We diluted 3.0 mL of raw effluent in 100 mL for the adsorption purposes. We found that the hydrogels adsorbed over 90% methylene blue and 62% DR from the effluent within a shorter period of time.

We further determined the effect of temperature, time and reaction environment on absorption. We found that a comparatively moderate range of temperature (25–35 °C), and time (2–3 h agitation) was highly favorable for the maximum adsorption of metal ions and dyes from the aqueous solution and effluent. From Figure 5d, it can be seen that hydrogels swelled less, possibly due to the fact that in hydrogel samples, metal ions might be clogged at the pores of the hydrogel, which leads to lower amounts of hydration of the hydrogel. In addition, metal ions might make a complex structure with the hydrogel functional groups in this process. On the other hand, when effluent was used, the hydrogel samples swelled to the maximum position compared to their metal ion absorption counterpart. This might be due to the fact that functional groups on the hydrogel backbone also helped in making hydrogen bonds with the pollutants [2,49].

## 3. Materials and Methods

### 3.1. Materials and Hydrogel Preparation

#### 3.1.1. Materials

Sugarcane bagasse was collected from Rajshahi Sugar Mills Ltd., Rajshahi, Bangladesh. It was sundried and then dried in an oven for 4 h at 105 °C. The dried bagasse was powdered by a crusher. The compositions of the sugarcane bagasse were as follows: 42.39% cellulose, 19.05% lignin, 28.68% hemicellulose and 9.88% other materials. All other chemicals (monomers-acrylamide (AM) and methyl methacrylate (MMA), initiator potassium persulphate (KPS, K_2_S_2_O_8_)) and crosslinking agent N,N-methylene-bis-acrylamide (MBA, CH_2_(H_2_C=CHCONH)_2_) used for preparation of the hydrogel were purchased. All the chemicals were analytical grade.

#### 3.1.2. Preparation of Bagasse-g-poly (AM-co-MMA) Hydrogels

**Scheme 1:**
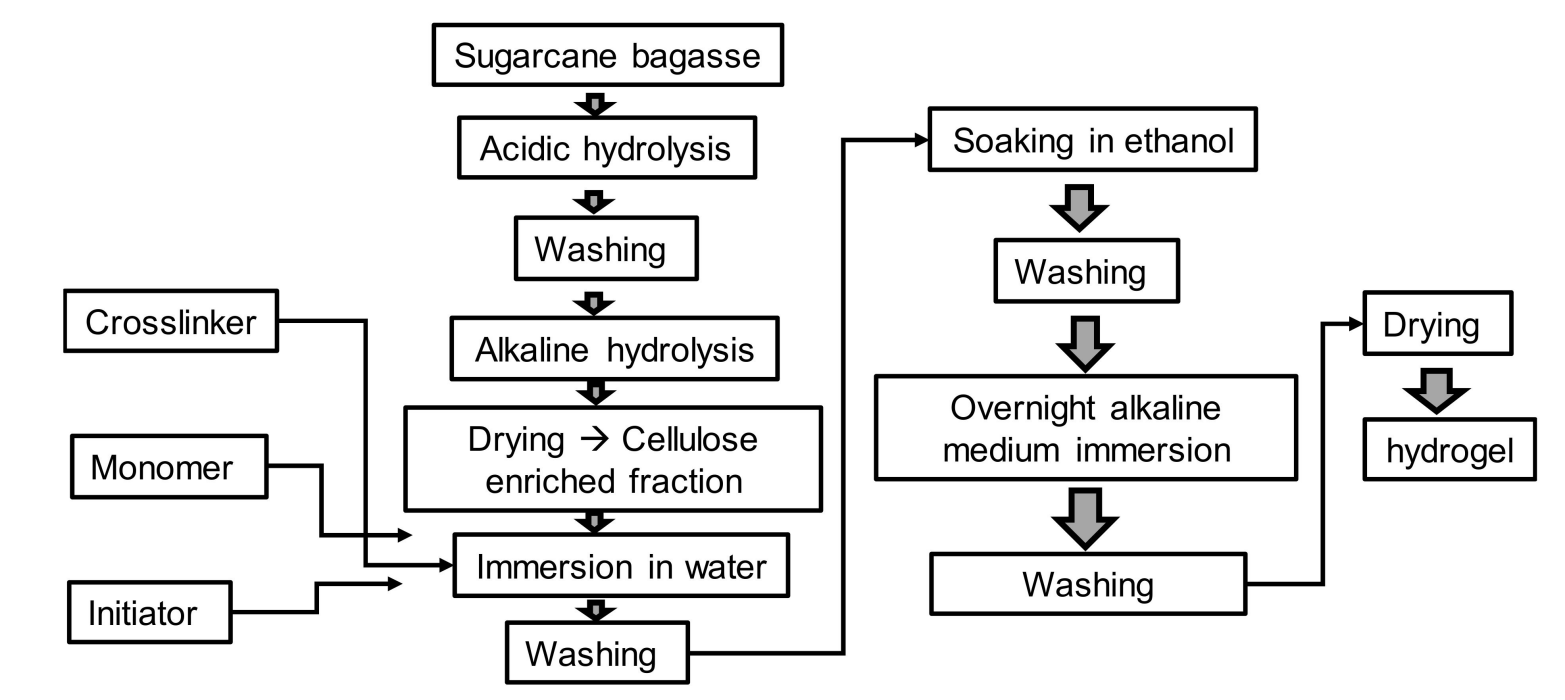
Schematic diagram of hydrogel preparation from sugarcane bagasse-based agro-waste.

Fresh and clean sugarcane bagasse was cut into small pieces and dried in sunlight for two days to reduce the moisture content. Then, the pieces were dried at 105 °C for 6 h in a Forced Convection Oven (FC-610, Toyo Seisakusho Co., Ltd., Hokkaido, Japan). Sugarcane bagasse (untreated bagasse, UB) was ground into powder using a disk mill (model: FFC-15, China). The sugarcane bagasse (1.0 g) was pre-hydrolyzed using sulfuric acid (15 mL, 1.5% *w*/*v*) at 100 °C for 3 h. The pre-hydrolyzed material was pulped using sodium hydroxide (17.5%) at 100 °C for 3 h, and the resulting pulp was filtered and washed with distilled water for several three times. When the pH reached 7, it was dried at ambient temperature to obtain treated bagasse, which is enriched with cellulose fractions. We termed this fraction as treated bagasse (TB). The compositions of TB were 47.6% cellulose, 24.4% hemicellulose, 18.5% lignin, 3.4% fatty and waxy materials, 2.5% ash and 3.6% others. Treated bagasse (TB)-based grafted hydrogels were synthesized by free radical graft copolymerization in the presence of an initiator KPS, monomer and crosslinking agent MBA. Overall process is shown in the Scheme 1.

In the hydrogel preparation, 0.5 g of treated bagasse was immersed in 15 mL of distilled water in a three-necked flask fitted with a magnetic stirrer, a reflux condenser and a nitrogen line at 50 °C. The solution was then bubbled with nitrogen for 30 min before starting the copolymerization reaction. Then, 0.05 g of KPS initiator (10% *w*/*w* basis of dried treated bagasse) was dissolved, and it was added to the bagasse-containing solution with stirring for 10 min to initiate radical formation. The total volume of the solution was controlled to 30 mL. Thereafter, monomers (1.25 g Acrylamide, AM and 1.175 g MMA) were added, and finally MBA was added. The mixture was then stirred for 30 min, and the reaction was continued for 2 h more at a temperature range of 60~80 °C. At the end of the reaction, the prepared hydrogel was removed carefully and washed with distilled water. Again, the hydrogels were washed several times with pure ethanol for dewatering purposes and immersed in 1.0 M NaOH solution for hydrolysis for 24 h. Finally, the hydrogel was washed with distilled water to remove additional NaOH and dried in a forced convection oven for 24 h at 60 °C [32]. Note that we did not prepare pure poly (AM-Co-MMA) hydrogel, as in the literature it showed that this pure hydrogel had lower water adsorption capacity (130%) [50].

### 3.2. Characterizations of Hydrogels

#### 3.2.1. Grafting Percentage, Water Absorbency, Effect of pH and Temperature of Hydrogels

Grafting percentage was calculated using weight change basis. Initially, the weight of cellulosic material was measured, and after completion of the polymer reaction and necessary treatment, the final weight of the sample was retaken. Then, grafting yield was calculated as grafting yield (%) = ((W*_f_* − W*_i_*)/W*_i_*) × 100, where W*_f_* and W*_i_* are the final and initial weight, respectively, of the hydrogel sample and bagasse sample.

Water absorbency is the fundamental property of hydrogels. For the measurement of water absorbency, a pre-weighed sample was immersed in distilled water for 12 h. Surface water was removed by gently dabbing with tissue paper, and then the sample was weighed again. The water absorbency was calculated using Equation (1) [33]:

[W*_eq_* = (M*_eq_* − M*_o_*)/M*_o_*] (1)

where M*_o_* is the weight of dry hydrogel in grams, M*_eq_* is the weight of wet hydrogel at equilibrium and W*_eq_* is the water absorbency in g/g.

Swelling behavior of the hydrogel samples was also determined at different pH levels in deionized water. For this purpose, buffer solution at a chosen pH was prepared, and water absorbency of hydrogels was calculated at that pH using Equation (1).

The water absorption capacity of synthesized hydrogels was also carried out at different temperatures, i.e., at 5 °C, 25 °C and 35 °C. The same procedures were followed as in the water absorbency measurement. The obtained data were plotted graphically to the determine the water retention capacity of the hydrogels.

#### 3.2.2. Determination of Gel Content

In general, the gel content of hydrogel signifies the amount of crosslinking that happened during the preparation of hydrogel. The greater the gel content, the higher the percentage of crosslinking; however, it may not ensure higher water absorbency. There should be a balance in gel content, i.e., crosslinking, to maintain higher water absorbency. For the determination of gel content, a fixed amount of hydrogel sample was dried to a constant weight and then immersed in 150 mL of distilled water for 24 h, with mild stirring to remove sol fractions. Then, the swollen samples were removed from the distilled water and dried to a constant weight again in an oven. The gel content was calculated according to Equation (2) [34]:

Gel content (%) = (W*_d_*/W*_i_*) × 100 (2)

where W*_d_* is the weight of dry gel after extraction in water and W*_i_* is the initial weight of dry gel.

#### 3.2.3. FTIR Analysis

FTIR analysis of the treated sugarcane bagasse and synthesized hydrogels was carried out using a FTIR spectrophotometer (Model: FTIR-8900, Shimadju, Kyoto, Japan) within the frequency range of 400 to 4000 cm^−1^. Samples were finely ground and mixed with KBr in the ratio of 1:100 and pressed to form a pellet suitable for FTIR spectra measurement.

#### 3.2.4. Surface Morphology and Crystallinity Analysis of Hydrogels

Surface morphology and crystallinity analysis of the prepared hydrogel samples was performed by Scanning Electron Microscope (SEM) and X-ray diffraction spectroscopy (XRD), respectively. The prepared hydrogel samples were dried at room temperature. Then, the surface morphologies of both raw and treated sugarcane bagasse and synthesized hydrogel (swelling state) were studied by SEM. As the bagasse and hydrogel are non-conductive, a carbon coating was applied (≈100 Å), and the sample was examined in an SEM chamber (Jeol, Model-JSM-7600F, Tokyo, Japan) to see the surface structures. The micrographs were enlarged to a magnification of 100 to 5000 times using (5–20 kV) accelerating voltage. XRD analysis was carried out with an X-ray diffractometer (Empyrean, PANalytical, Almelo, The Netherlands). The necessary specifications of the XRD instrument are as follows: X-ray source was Cu-K*α*, tube voltage was 45 kV, and electric current was 40 mA. Before measurement, the samples for XRD of hydrogel were transformed to dried powder form. All the samples were transformed to powder form using a mortar and pestle. The well-ground sample had a particle size of, at most, 44 microns and was dried/dehydrated using a drying oven.

#### 3.2.5. Thermal Analysis of Sample

Thermogravimetric analysis (TGA) of the samples was carried out using TGA equipment equipped with DTA/DSC (NETZSCH STA 449F3, Selbe, Germany). The relevant mass loss rate with relative temperature (DTA/DSC curves) was determined to observe the nature of the reaction, i.e., whether the reaction was exothermic or endothermic. The tests were conducted between 0 and 800 °C under an inert atmosphere (nitrogen). The heating rate and the air flow rate were 20 °C/min and 200 mL/min, respectively.

### 3.3. Biodegradation and Water Retention Capacity of Hydrogels

The biodegradation test was carried out using a simple method of dipping the hydrogel in soil for a period of time. The change of residual mass was measured, and the degradation percentage was calculated. For this reason, different sacrificial samples were prepared, and biodegradation percentage was calculated.

The water retention capacity of the hydrogels was determined by placing the samples at different temperatures and leaving them up to 50 h. The samples were reweighed to determine the weight differences at different time intervals. Initial water absorption capacity was determined using Equation (1). The remaining calculations were carried out based on percentage basis.

### 3.4. Inorganic and Organic Ion Adsorption Capacities of Hydrogels

A total of 100 mL aqueous solution containing 50 ppm of each inorganic ion, such as Cu^2+^, Fe^2+^ and Cr^3+^ ions, and organic dyes such as methylene blue and drimerin red was prepared for the adsorption experiment to compare the performance of hydrogels. Then hydrogel samples were immersed in the solutions, and the solutions were stirred at 100 rpm for 3 h so that the hydrogels could adsorb metal ions and dyes from the aqueous solutions [35,36,37,38]. The swollen samples of hydrogel were separated by filtration. The concentrations of the residual metal ions were determined by atomic absorption spectrometry (AAS), and the concentrations of the dyes were determined using a UV-visible spectrophotometer. The amount of metal ions or dye adsorbed by the hydrogel was determined by Equation (3), as follows:

(3) $$Qe\;=\;\frac{\left(C_{o}-C_{e}\right)\;\times \;V}{W\;}$$

where *C_o_* is the initial concentration of dyes or metal ions, C*_e_* is the equilibrium concentration after adsorption by the hydrogels, V is the total volume (L) of the solution and W is the weight of the dry hydrogel.

## 4. Conclusions

An agro-waste-based superabsorbent hydrogel was synthesized using a simple and energy efficient grafting copolymerization technique from treated sugarcane bagasse with an acrylamide and methyl methacrylate monomer. The characterization study indicated that the prepared hydrogel was a porous structure (SEM analysis) and amorphous oriented (XRD pattern). Influential factors such as temperature, pH and concentrations were controlled achieve a higher swelling ratio of the hydrogels. Instrumental analysis indicated that the polymerization was complete, and a stable hydrogel structure was produced. The hydrogel can degrade in the natural environment and showed good swelling (28 g/g) and water retention capacity (about 50 h). The super-absorption capacity of the hydrogel made it an effective adsorbent to remove many inorganic ions as well as organic dyes. While we assume this material will have versatile application, it requires further study.

## Figures and Tables

**Table 1:** Determination of gel fraction in hydrogel.

No. of Sample	Initial Weight of Dry Gel, W*_i_* (g)	Weight of Extracted Dry Gel, W*_d_* (g)	Gel Fraction (%)	Average Percentage of Gel Fraction (%)
01	0.163	0.147	90.2	90.1
02	0.149	0.135	90.6	
03	0.144	0.129	89.6	

## Data Availability

Data are contained within the article.
